# Intra and interobserver agreement of the Dynamic Imaging Grade of Swallowing Toxicity Scale (DIGEST) in fiberoptic endoscopic evaluation of swallowing (FEES): the importance of observer-tailored training

**DOI:** 10.1007/s00405-023-07840-1

**Published:** 2023-01-27

**Authors:** Sorina R. Simon, Monse W. M. Wieland, Charlotte Hendriks, Walmari Pilz, Antonio Schindler, Bjorn Winkens, Laura W. J. Baijens

**Affiliations:** 1 Department of Otorhinolaryngology, Head and Neck Surgery, Maastricht University Medical Center, P.O. Box 5800, 6202 AZ Maastricht, The Netherlands; 2 Department of Radiology and Nuclear Medicine, Maastricht University Medical Center, Maastricht, The Netherlands; 3 GROW-School for Oncology and Reproduction, Maastricht University Medical Center, Maastricht, The Netherlands; 4 School for Mental Health and Neuroscience-MHeNs, Maastricht University Medical Center, Maastricht, The Netherlands; 5 Department of Biomedical and Clinical Sciences “L. Sacco”, University of Milan, Milan, Italy; 6 Department of Methodology and Statistics, Maastricht University, Maastricht, The Netherlands; 7 Care and Public Health Research Institute-CAPHRI, Maastricht University Medical Center, Maastricht, The Netherlands

**Keywords:** Head and neck cancer, Dysphagia, Deglutition, Swallowing assessment, Fiberoptic endoscopic evaluation of swallowing

## Abstract

**Purpose:**

The Dynamic Imaging Grade of Swallowing Toxicity (DIGEST) is a scale to quantify the severity of pharyngeal dysphagia in head and neck cancer (HNC) patients. This study (1) described the training process of the observers for DIGEST in fiberoptic endoscopic evaluation of swallowing (FEES), (2) determined observer agreement on the DIGEST in FEES, (3) explored the effect of bolus consistency on observer agreement, and 4) explored criterion validity of the DIGEST in FEES.

**Methods:**

Twenty-seven dysphagic HNC patients were enrolled. Two observers completed a training program for DIGEST in FEES. Observer agreement on the Penetration-Aspiration Scale (PAS), percentage of pharyngeal residue (PPR), and DIGEST grades was determined using linearly weighted Cohen’s kappa coefficient (*κ*).

**Results:**

Due to insufficient observer agreement after the first measurement attempt, additional training was organized using an elaborated manual with descriptions of the visuoperceptual variables, thereby improving observer agreement. Intraobserver agreement was almost perfect on the PAS (*κ* = 0.86–0.88) and PPR (*κ* = 0.84–0.86). Interobserver agreement was substantial on the PAS (*κ* = 0.78), almost perfect on the PPR (*κ* = 0.82), substantial on the safety grade (*κ* = 0.64), almost perfect on the efficiency grade (*κ* = 0.85), and substantial on the summary grade (*κ* = 0.71). Bolus consistency had an effect on observer agreement. A significant correlation was found between DIGEST efficiency grade and EAT-10.

**Conclusion:**

The DIGEST showed to be a reproducible measurement for FEES in terms of observer agreement. However, agreement between novice observers on the DIGEST was only reached after specific observer-tailored training. Observer agreement should be analyzed by taking bolus consistency into account during training, as this might affect the interpretation of the outcome. A manual with well-defined descriptions can optimize the reproducibility of DIGEST measurements.

**Supplementary Information:**

The online version contains supplementary material available at 10.1007/s00405-023-07840-1.

## Introduction

Patients with head and neck cancer (HNC) often experience pharyngeal dysphagia, which can be caused by the cancer itself and/or by the oncological treatment [1, 2]. An accurate evaluation of swallowing function is paramount to guide dysphagia management. Videofluoroscopic swallowing study (VFSS) and fiberoptic endoscopic evaluation of swallowing (FEES) are widely considered gold standards for the instrumental assessment of swallowing [3–5]. During VFSS or FEES swallowing safety (penetration or aspiration) [6–9] and swallowing efficiency (pharyngeal residue) [9–12] can be measured. These measurements are carried out by observers and are based on subjective judgement [9, 13, 14]. As VFSS and FEES are completely different imaging techniques, observers have a different perspective when measuring the same variables [6, 10, 15]. To date, only few visuoperceptual measurement scales for VFSS and FEES have been validated [7, 8, 16, 17]. The Dynamic Imaging Grade of Swallowing Toxicity (DIGEST) was developed for grading the overall severity of pharyngeal dysphagia in HNC patients before or after oncological treatment [18]. The DIGEST was initially developed and validated for VFSS. Recently, this scale was validated for FEES by Starmer et al. [19]. Measurement scales such as the Penetration-Aspiration Scale (PAS) and percentage of pharyngeal residue (PPR) measure only one specific aspect of swallowing, thus these scales cannot determine overall dysphagia severity if used as the sole measurement. The DIGEST, however, uses the integration of the aforementioned phenomena of swallowing safety (penetration and/or aspiration) and efficiency (pharyngeal residue) to arrive at a composite severity score for pharyngeal dysphagia [18]. A reproducible measurement scale for the severity of dysphagia is very valuable for clinical practice as decision making on dysphagia treatment is, among others, based on the results of these measurements. However, observer agreement has an impact on reproducibility and on the validity of a test because if the observers who perform the measurements, cannot agree on the values after measuring the same variables, the test results will be of little use. Interobserver agreement refers to the degree to which two or more independent observers report the same observed values after measuring the same variables. An accurate diagnosis, sensitivity, specificity, predictive values, and likelihood ratios are items that address the validity of a test [20]. However, studies on FEES with a detailed description of the training process of observers to obtain sufficient intra and interobserver agreement on visuoperceptual measurements are scarce [11, 21].

Currently, there is very little evidence in the literature with regard to the reproducibility and external validity of the DIGEST in FEES, as only one study investigated these methodological aspects [19]. Additional research is required to assess the methodological robustness of the DIGEST measurements in FEES, and studies among different study populations can also contribute to improve external validity. In Europe, different health professionals often being member of an interdisciplinary dysphagia team may use the DIGEST, including speech–language pathologists, laryngologists, physician assistants, occupational therapists, etc. This wider use by multiple professionals underlines the importance of increasing our understanding of the conditions and restrictions of the reproducibility of the DIGEST in FEES. The present study investigated how to reach agreement among observers on the DIGEST in FEES to increase the body of evidence in the literature.

The study aims to (1) describe the training process of the observers for DIGEST in FEES, (2) determine observer agreement on the DIGEST in FEES, (3) explore the effect of bolus consistency on observer agreement, and (4) explore the criterion validity of the DIGEST in FEES. It is hypothesized that the DIGEST is a reproducible measurement for FEES in terms of observer agreement. Moreover, it is expected that observer agreement of novice observers will improve after completion of a training program.

## Methods

### Study design and patient selection

For this cross-sectional study, HNC patients who underwent a standardized FEES examination between June 2016 and October 2020 in the interdisciplinary outpatient clinic for dysphagia of the Comprehensive Cancer Center of Maastricht University Medical Center in the Netherlands were included. Exclusion criteria were: a history of total laryngectomy or total glossectomy, a Mini Mental State Examination score below 23, not being able to tolerate or handle more than one bolus consistency during FEES, and any concurrent diagnosis causing dysphagia (stroke, Parkinson's disease, cervical spine surgery, dementia, etc.) [22]. Data on demographic patient characteristics, tumor staging, and oncological treatment were collected according to the Dutch Head and Neck Audit (DHNA) [23] and retrospectively extracted from the electronic health records. Cancer staging was carried out according to the tumor, nodes, and metastasis classification (TNM classification, 8^th^ edition) [24]. The study protocol was approved by the medical ethics committee (METC 2020–1321) and all patients gave their informed consent.

### Swallowing assessment

All patients underwent a standardized swallowing assessment, including a clinical ear, nose, and throat examination, the Functional Oral Intake Scale (FOIS), the Eating Assessment Tool (EAT)-10, the MD Anderson Dysphagia Inventory (MDADI), and a standardized FEES examination.

The FOIS is a clinician-reported scale to determine the level of oral intake of food and liquids in dysphagic patients [25]. This ordinal scale ranges from 1 to 7 where level 1 represents tube feeding dependency and level 7 represents a total oral diet without any restrictions [25].

The EAT-10 is a patient-reported 10-item dysphagia-specific symptom questionnaire and the Dutch version was completed by all the patients [26, 27]. An EAT-10 ≥ 3 score is considered abnormal and represents a higher level of self-perceived symptom severity [26].

The Dutch version of the MDADI was also completed [28–30]. The MDADI is a patient-reported 20-item dysphagia-specific quality-of life (QoL) questionnaire that consists of 4 subscales (global, functional, physical, and emotional subscale). Responses are summed to calculate the total MDADI score (MDADI-T): a minimum score of 20 represents a poor dysphagia-specific QoL whereas a maximum score of 100 represents a high dysphagia-specific QoL.

During the FEES examination, the following standardized protocol was carried out: three boluses of thin liquid (3 × 10 cc water), three boluses of thick liquid (3 × 10 cc applesauce; ‘One2fruit’), and one bite-sized cracker (Delhaize mini toast 80 g). Each liquid bolus was dyed with 5% methylene blue to enhance endoscopical visualization [11, 31, 32]. The viscosities of thin and thick liquid boluses were, respectively, 1 mPa s for thin liquid and 1200 mPa s for thick liquid. The viscosities were measured at 25 °C and 50 s^−1^ of shear rate as recommended by the National Dysphagia Diet [33]. According to the International Dysphagia Diet Standardisation Initiative (IDDSI), thin liquid was classified as IDDSI level zero ‘thin’ and thick liquid as IDDSI level 3 ‘moderately thick’ during the flow test [34]. The position of the tip of the flexible endoscope (Pentax FNL-10RP3, Pentax Canada Inc., Mississauga, Ontario, Canada) ensured observation of the pharyngolaryngeal anatomy and physiology during swallowing. Topical anesthetics, which may affect pharyngolaryngeal sensory function, were not applied. FEES videos were recorded on a secured network drive of the hospital at 25 frames per second using a Xion SD camera, XionEndoSTROB E camera control unit and Matrix DS data station with DIVAS software (Xion Medical, Berlin, Germany).

The seven bolus swallows of each patient were split in seven separate video clips. The clips of all the patients were pseudonymized and randomized prior to the measurement process. The observers were blinded to the order of the bolus swallows, patient’s identity and clinical data, and to each other’s measurements. During the measurement process, the FEES video clips were analyzed at varying speed (normal to frame-by-frame) using Quick Time Media Player (Apple Inc, Cupertina, California, USA) and repeated as often as necessary. Observers were instructed to limit the duration of each session to two hours, to avoid attentional bias due to fatigue. To obtain intraobserver agreement, each observer repeated the same measurements again blinded and in randomized order. These measurements were performed with an interval of at least one week to avoid memory bias.

### DIGEST

The DIGEST is based on the integration of two primary outcome measurements representing swallowing safety and swallowing efficiency [18]. The DIGEST safety grade is based on the maximum score of the PAS over all bolus swallows [8]. The PAS is a well-known 8-point ordinal scale to measure the severity of airway invasion by the bolus. The maximum PAS score is then transferred into one of the four pooled PAS categories: PAS 1–2, PAS 3–4, PAS 5–6, and PAS 7–8. Thereafter, modifiers are applied to account for the amount and frequency or pattern of penetration/aspiration events. After applying the modifiers, a safety grade is determined (grade 0–4).

The DIGEST efficiency grade is based on the maximum score of the PPR over all bolus swallows. The PPR after the first swallowing movement per bolus (so without clearing swallows on that single bolus) is measured. The maximum PPR score is then transferred into one of the four residue categories: < 10%, 10–49%, 50–90%, and > 90%. Thereafter, again modifiers are applied to account for variations across different bolus consistencies. After applying these modifiers, an efficiency grade is determined (grade 0–4) [18].

For each patient, an overall pharyngeal dysphagia severity grade (the summary DIGEST grade, ranging from 0 to 4) is obtained by the integration of the safety and efficiency grade according to the DIGEST safety and efficiency profiles table of the DIGEST study in VFSS [18]. DIGEST grade 0 represents no pharyngeal dysphagia, grade 1 mild, grade 2 moderate, grade 3 severe, and grade 4 life-threatening pharyngeal dysphagia [18].

### Training process

Two novice observers (Master of Medicine students) without previous experience in swallowing assessment followed an intensive training on the measurement of the PAS and PPR in FEES videos. Master of Medicine students who participate in the 4-month fulltime mandatory scientific internship and write a scientific master thesis are in their final year of the Master of Medicine. In this final year they also did a 6-month fulltime clinical internship in the department of otorhinolaryngology, working under supervision on the hospitalization ward and in the outpatient clinic having three new patients daily to examine (including flexible endoscopy) under supervision. The reason for selecting novice observers was based on the fact that these observers will pose a bigger challenge in using the DIGEST in terms of reproducibility of measurements compared to experienced clinicians.

The training process is presented in a flowchart in the supplementary information (Online Resource 1). The duration of the training sessions was approximately one hour, interspersed with homework assignments. The training was given by an expert clinician (speech-language pathologist W.P.) with more than 10 years of clinical and scientific experience in performing and interpreting FEES examinations.

During the training, the novice observers were educated about the anatomy and physiology of the pharynx and larynx and about the purpose and protocols of the FEES examination using FEES sample videos for demo purpose. Thereafter, the observers received instructions on the interpretation of the definitions of the PAS and PPR categories and how to measure these variables. The definitions of the variables were explained verbally using visual depictions of the ordinal categories of both variables. When the observers understood the definition of the ordinal variables, the FEES variables were scored by the expert clinician in the presence of the observers.

Seven joint training sessions were held, in which the PAS and PPR variables were reviewed and scored by the observers under supervision of the expert clinician. After each training session, the observers received a batch of 10 to 40 FEES video clips that should be scored independently as homework assignments. In the next training session, the results of the homework assignments were reviewed and revised if necessary. Any disagreement in the scores was discussed with the expert clinician and a consensus on the interpretation of the variables was reached.

A written manual containing definitions of the ordinal variables, including points-of-attention from the analysis of disagreements during the training program, was developed. This user manual was available for the observers during the subsequent measurements of the experiment. The training sessions were completed when the observers reached a percentage of agreement > 70% and felt confident to start measuring the variables in FEES video clips for the present experiment.

As observer agreement was not sufficient after the first measurement attempt of the experiment, the observers underwent an additional training program. This was done to identify and understand reasons for disagreement and subsequently reach consensus to improve observer agreement during the second measurement attempt.

### Statistical analysis

Normally distributed baseline characteristics were represented by means and standard deviation (SD). Median and interquartile range (25th and 75th percentile) were used to describe baseline characteristics when the frequency distribution of the data was skewed. Normality was assessed using histograms and Q-Q plots. Frequencies and proportions were used for ordinal variables. Intra and interobserver agreement were calculated using linearly weighted Cohen’s kappa coefficient (*κ*) and percentage of agreement. The linearly weighted kappa was interpreted as follows: < 0 no agreement, 0.01–0.20 slight agreement, 0.21–0.40 fair agreement, 0.41–0.60 moderate agreement, 0.61–0.80 substantial agreement, and 0.81–1.00 almost perfect agreement [35]. An agreement of ≥ 0.61 was considered sufficient. To explore the criterion validity of the DIGEST in FEES, the correlation between safety grade, efficiency grade, and summary DIGEST grade versus the EAT-10, FOIS, TNM, and MDADI (MDADI-T and subscales, including global, functional, physical, and emotional subscale) was determined using Kendall’s Tau-b correlation coefficient. All statistical analyses were performed using IBM SPSS Statistics for Windows, version 25 (IBM, Armonk, NY).

## Results

### Patient characteristics

Twenty-seven HNC patients were included in this study. The mean age of the patients was 64.1 years (SD 9.1). The majority of the patients were male (*N* = 20) (74.1%). Five patients underwent pre-treatment FEES evaluations (18.5%). The median score (25th–75th percentile) of the FOIS was 5 (5–6). Patient characteristics are presented in Table 1.

**Table 1:** Frequency distributions of data on tumor staging characteristics, oncological treatment, FOIS, and dysphagia-specific questionnaires (total number of HNC patients = 27)

Variables	Number of patients = 27
Tumor site	*N* (%)
Pharynx	14 (51.9)
Oral cavity	5 (18.5)
Larynx	7 (25.9)
Unknown primary tumor	1 (3.7)
T classification	*N* (%)
T0–1	7 (25.9)
T2	12 (44.4)
T3	5 (18.5)
T4	3 (11.1)
N classification	*N* (%)
N0	9 (33.3)
N1	3 (11.1)
N2	13 (48.1)
N3	2 (7.4)
M classification	*N* (%)
M0	23 (85.2)
M1	4 (14.8)
Treatment modality	*N* (%)
Surgery	2 (7.4)
Surgery and adjuvant (chemo)radiation	4 (14.8)
Primary (chemo)radiation	21 (77.8)
Radiation characteristics	
Mean total dose in Gray (SD)	67.6 (6.5)
Mean number of fractions (SD)	33.6 (4.2)
FOIS	*N* (%)
1 (nothing by mouth)	0 (0)
2 (tube dependency with minimal attempts of food or liquid)	0 (0)
3 (tube dependency with consistent oral intake of food or liquid)	1 (3.7)
4 (total oral diet of a single consistency)	0 (0)
5 (total oral diet with multiple consistencies requiring special preparation or compensations)	17 (63.0)
6 (total oral diet with multiple consistencies without special preparation, but with specific food limitation)	3 (11.1)
7 (a total oral diet without any restrictions)	6 (22.2)
	Median (25th–75th percentile)
BMI	22.8 (20.4–28.9)
Time interval in months between end of oncological treatment and FEES (N = 22)	21.1 (4.6–76.2)
	Mean (SD)
EAT-10	14.2 (10.3)
MDADI	
Total score	66.8 (17.5)
Global subscale score	3.0 (1.4)
Functional subscale score	19.5 (4.4)
Physical subscale score	23.1 (9.1)
Emotional subscale score	19.3 (6.6)

*HNC* head and neck cancer, *N* number of patients, *SD* standard deviation, *FOIS* Functional Oral Intake Scale, *FEES* fiberoptic endoscopic evaluation of swallowing, *EAT-10* The Eating Assessment Tool, *BMI* body mass index, *MDADI* MD Anderson Dysphagia Inventory

### First measurement attempt

During the first measurement attempt of the observers, the PAS and PPR were measured in 78 randomized bolus swallows of 27 HNC patients. To obtain intraobserver agreement, each observer repeated the same measurements in the 78 randomized bolus swallows with an interval of at least one week and again blinded. Observer agreement during the first measurement attempt of the present experiment is presented in Table 2.

**Table 2:** Linearly weighted kappa coefficient and percentage of agreement on the PAS and PPR when considering all bolus consistencies together (‘total’) and per bolus consistency during the first measurement attempt^a^

FEES variable and bolus consistency	Intraobserver agreement					Interobserver agreement		
	*N* (%)	Observer 1		Observer 2		*N* (%)	Kappa (SE)	%
		Kappa (SE)	% of agreement	Kappa (SE)	% of agreement			of agreement
First measurement attempt								
PAS								
Total	78	0.90 (0.04)	87.2	0.87 (0.04)	82.9	78	0.77 (0.08)	71.8
Thin liquid	33 (42)	0.91 (0.04)	82.4	0.86 (0.05)	69.7	33 (42)	0.72 (0.20)	60.6
Thick liquid	32 (41)	0.88 (0.07)	90.9	0.84 (0.07)	90.3	32 (41)	0.78 (0.09)	74.2
Bite-sized cracker	13 (17)	^b^	90.9	1.00 (0.00)	100	13 (17)	1.00 (0.00)	100
PPR								
Total	78	0.85 (0.06)	92.3	0.59 (0.09)	77	78	0.62 (0.09)	78.7
Thin liquid	33 (42)	0.88 (0.12)	96.3	0.67 (0.13)	80	33 (42)	0.38 (0.15)	62.5
Thick liquid	32 (41)	0.82 (0.09)	89.3	0.50 (0.15)	73.1	32 (41)	0.87 (0.09)	96.3
Bite-sized cracker	13 (17)	0.74 (0.24)	90	0.54 (0.26)	80	13 (17)	0.58 (0.26)	80

*PAS* Penetration-Aspiration Scale [8]; *PPR* percentage of pharyngeal residue; *FEES* fiberoptic endoscopic evaluation of swallowing; *N* number of bolus swallows; *SE* standard error

^a^Following the initial training program on the measurement of the PAS and PPR in FEES, a first measurement attempt was made in which the PAS and PPR were measured in 78 bolus swallows of 27 HNC patients (Table 2). Due to unexpectedly low observer agreement (especially regarding PPR), an additional training program was organized. During the second measurement attempt, the PAS and PPR were measured in 184 bolus swallows of 27 HNC patients (Tables 3, 4)

^b^Linearly weighted kappa could not be carried out for all measurements due to a limited number of measurements for some bolus consistencies, such as for bite-sized cracker, or a lack of variation of the scores across the PAS scales

Linearly weighted kappa coefficient could not be carried out for all measurements due to a limited number of measurements for some bolus consistencies or a lack of variation of the scores across the PAS or PPR scales. For example, a limited number of measurements for bite-sized cracker was obtained due to the lower number of HNC patients who were able to process this consistency because of severe xerostomia. In case of lack of variation of scores, the kappa may incorrectly conclude that the agreement is low (as the correction for chance is too strict). To check whether this was the case, percentage of agreement as a measure of intra and interobserver agreement was also calculated for all bolus consistencies. This limitation of linearly weighted kappa coefficient as measure of agreement is further explained in the Discussion section.

#### Penetration-Aspiration Scale

Intraobserver agreement (overall and per bolus consistency) of both observers on the measurement of the PAS was sufficient (*κ* ≥ 0.84) (Table 2). Interobserver agreement (overall and per bolus consistency) on the PAS was sufficient too (*κ* ≥ 0.72).

#### Percentage of pharyngeal residue

The overall intraobserver agreement of both observers on the measurement of the PPR showed notable variation, when considering all bolus consistencies together (*κ* = 0.59–0.85) (Table 2). Observer 1 presented substantial to almost perfect intraobserver agreement for all measurements. Observer 2 did not reach sufficient intraobserver agreement for both thick liquid and bite-sized cracker when agreement was calculated using linearly weighted kappa coefficient (*κ* ≤ 0.60). However, the corresponding percentage of agreement was 73.1% for thick liquid and 80% for bite-sized cracker.

The overall interobserver agreement was substantial, when considering all bolus consistencies together (*κ* = 0.62). Interobserver agreement was not sufficient for thin liquid (*κ* = 0.38) and bite-sized cracker (*κ* = 0.58) using linearly weighted kappa coefficient, whereas percentage of agreement was 62.5% for thin liquid and 80% for bite-sized cracker.

### Additional training program

In the attempt to improve observer agreement, an additional training program consisting of three training sessions was organized in a period of four weeks. Again, the expert clinician and the observers measured the FEES variables in several FEES sample videos together, exploring the reasons of disagreement between the observers. Specific attention was paid to variables with insufficient interobserver agreement (*κ* ≤ 0.60) per bolus consistency during the first measurement attempt, in particular PPR. As PPR is based on a continuous scale (0–100%), the categorization of this continuous variable into an ordinal scale variable seems to be based on arbitrary cut-off values, and to distinguish between a PPR of 49% (category 10–49%) and a PPR of 50% (category 50–90%) is not an easy task. During this additional training, the written user manual containing the definitions of the variables was further improved by revising and adjusting the descriptions and range of each level of the PPR measurement scale per bolus consistency. Points-of-attention discussed during this additional training program and corresponding images of severity levels of pharyngeal residue were added to the user manual. Thereafter, the manual was further revised and optimized by two expert clinicians. The procedure of this expert revision consisted of two sessions in which the expert clinicians discussed the corresponding images of the severity levels of the PPR. In between these sessions, the expert clinicians studied the advantages and disadvantages of the descriptions and corresponding images independently, and in the second session the expert clinicians made a final consensus decision on the selection of the corresponding images. This expert opinion was determined as ‘gold standard’. This manual with well-defined descriptions was used as a reference to enhance the agreement within and between observers during the second measurement attempt.

### Second measurement attempt

During the second measurement attempt, the PAS and PPR were measured in 184 randomized bolus swallows of the same 27 HNC patients. To obtain intraobserver agreement, each observer repeated the same measurements with an interval of at least one week, again blinded in a random selection of 59 out of 184 randomized bolus swallows. Frequency distributions of the scores of the PAS, PPR, DIGEST profile, and summary DIGEST grade given by each observer are presented in Table 3. Observer agreement on the PAS and PPR is presented in Table 4.

**Table 3:** Frequency distributions of the scores of the PAS, PPR, DIGEST profile, and summary DIGEST grade by each observer during the second measurement attempt (in total 184 bolus swallows of 27 HNC patients)

	Observer 1, *N* (%)	Observer 2, *N* (%)
PAS scores (*N* = 184 bolus swallows)		
1	87 (48.9)	73 (40.2)
2	36 (20.2)	54 (29.7)
3	23 (12.9)	29 (15.9)
4	5 (2.8)	3 (1.6)
5	6 (3.4)	6 (3.3)
6	8 (4.5)	4 (2.2)
7	8 (4.5)	7 (3.8)
8	5 (2.8)	6 (3.3)
Missing	6	2
PPR scores (*N* = 184 bolus swallows)		
1	83 (54.2)	92 (55.1)
2	57 (37.3)	54 (32.3)
3	11 (7.2)	18 (10.8)
4	2 (1.3)	3 (1.8)
Missing	31	17
DIGEST profile (*N* = 27 patients)		
S0E0	3 (11.1)	3 (11.1)
S1E0	2 (7.4)	3 (11.1)
S0E1	5 (18.5)	5 (18.5)
S1E1	6 (22.2)	6 (22.2)
S0E3	1 (3.7)	1 (3.7)
S1E3	2 (7.4)	6 (22.2)
S2E3	2 (7.4)	0 (0.0)
S3E0	1 (3.7)	1 (3.7)
S3E1	3 (11.1)	0 (0.0)
S3E3	2 (7.4)	2 (7.4)
Summary DIGEST grade (*N* = 27 patients)		
0	3 (11.1)	3 (11.1)
1	13 (48.1)	14 (51.9)
2	3 (11.1)	7 (25.9)
3	8 (29.6)	3 (11.1)
4	0 (0.0)	0 (0)

*PAS* Penetration-Aspiration Scale [8], *PPR* percentage of pharyngeal residue, *DIGEST* Dynamic Imaging Grade of Swallowing Toxicity, *HNC* head and neck cancer, *N* number of bolus swallows or number of patients (as specified in the table), *S* safety grade, *E* efficiency grade

**Table 4:** Linearly weighted kappa coefficient and percentage of agreement on the PAS and PPR when considering all bolus consistencies together (‘total’) and per bolus consistency during the second measurement attempt

FEES variable and bolus consistency	Intraobserver agreement					Interobserver agreement		
	*N* (%)	Observer 1		Observer 2		*N* (%)	Kappa (SE)	%
		Kappa (SE)	% of agreement	Kappa (SE)	% of agreement			of agreement
Second measurement attempt								
PAS								
Total	59	0.88 (0.05)	86.4	0.86 (0.07)	91.5	184	0.78 (0.04)	78.7
Thin liquid	26 (44)	0.83 (0.08)	76.9	0.77 (0.12)	84.6	79 (43)	0.82 (0.05)	76.3
Thick liquid	24 (41)	0.92 (0.06)	91.7	0.97 (0.03)	95.8	77 (42)	0.80 (0.05)	79.7
Bite-sized cracker	9 (15)	^a^	100	1.00 (0.00)	100	28 (15)	0.44 (0.13)	82.1
PPR								
Total	59	0.84 (0.08)	91.7	0.86 (0.07)	92.3	184	0.82 (0.04)	88.1
Thin liquid	26 (44)	0.78 (0.14)	89.5	0.92 (0.08)	95.7	79 (43)	0.84 (0.07)	91.9
Thick liquid	24 (41)	0.84 (0.11)	90.5	0.84 (0.11)	90.5	77 (42)	0.82 (0.06)	83.9
Bite-sized cracker	9 (15)	^a^	100	^a^	87.5	28 (15)	0.55 (0.18)	88.9

*PAS* Penetration-Aspiration Scale [8], *PPR* percentage of pharyngeal residue, *FEES* fiberoptic endoscopic evaluation of swallowing, *N* number of bolus swallows, *SE* standard error

^a^Linearly weighted kappa could not be carried out for all measurements due to a limited number of measurements for some bolus consistencies, such as for bite-sized cracker, or a lack of variation of the scores across the PAS or PPR scales

#### Penetration-Aspiration Scale

Intraobserver agreement (overall and per bolus consistency) of both observers on the measurement of the PAS was sufficient (*κ* ≥ 0.77) (Table 4). The overall interobserver agreement on the PAS was substantial, when considering all bolus consistencies together (*κ* = 0.78), showing improvement compared to the ‘first measurement attempt’. The lowest interobserver agreement was obtained for bite-sized cracker (*κ* = 0.44) using linearly weighted kappa coefficient. However, when looking at the percentage of agreement among the different bolus consistencies, the interobserver agreement for bite-sized cracker was 82.1%.

#### Percentage of pharyngeal residue

The overall intraobserver agreement of both observers on the measurement of the PPR was almost perfect, when considering all bolus consistencies together (*κ* = 0.84–0.86) (Table 4). The lowest intraobserver agreement was obtained for thin liquid for observer 1 (*κ* = 0.78). The overall interobserver agreement on the PPR was almost perfect, when considering all bolus consistencies together (*κ* = 0.82), showing improvement compared to the ‘first measurement attempt’. The lowest interobserver agreement was obtained for bite-sized cracker (i.e. moderate agreement) (*κ* = 0.55) using linearly weighted kappa coefficient. However, the corresponding percentage of agreement (88.9%) was similar to the other bolus consistencies.

### Observer agreement on safety, efficiency, and summary DIGEST grade

Based on the scores of the second measurement attempt, the observers independently determined the safety and efficiency grades, per patient, by applying the modifiers described in the DIGEST validation study for VFSS [18]. Interobserver agreement, presented in Table 5, was substantial to almost perfect (safety grade: *κ* = 0.65 (SE 0.12); efficiency grade: *κ* = 0.85 (SE 0.09)). The interobserver agreement on the summary DIGEST grade was substantial (*κ* = 0.71 (SE 0.09)).

**Table 5:** Interobserver agreement on the safety, efficiency, and summary DIGEST grade

Grade	Interobserver agreement	
	Linearly weighted kappa (SE)	% of agreement
Safety grade	0.65 (0.12)	74.1
Efficiency grade	0.85 (0.09)	88.9
Summary DIGEST grade	0.71 (0.09)	25.9

*DIGEST* Dynamic Imaging Grade of Swallowing Toxicity, *SE* standard error

### Criterion validity

To further explore the criterion validity of the DIGEST, the correlation between safety, efficiency, and summary DIGEST grade versus the EAT-10, FOIS, TNM, and MDADI (MDADI-T and subscales, including global, functional, physical, and emotional) was analyzed. No significant correlation was found between safety, efficiency, and summary DIGEST grade versus FOIS, TNM, and MDADI. However, the efficiency grade significantly correlated with the EAT-10 for both observers (observer 1: *p* = 0.01; observer 2: *p* = 0.008). Also, a significant correlation was found between the summary DIGEST grade and the EAT-10 only for the scores of observer 1 (*p* = 0.04), but not for the scores of observer 2 (*p* = 0.08). No significant correlation was found between the safety grade and the EAT-10.

## Discussion

The present study described the training process of two novice observers to obtain observer agreement on the visuoperceptual measurements of the DIGEST in FEES including effects of bolus consistency on agreement and statistical analysis to interpret the results. The development and implementation of a user manual with well-defined descriptions, in combination with a learning curve of the observers due to repeated training, led to a significantly better reproducibility of the DIGEST measurements in the present study. The criterion validity of the DIGEST was also explored using several explanatory variables (the EAT-10, FOIS, TNM, and the MDADI) to predict the DIGEST outcome. As our study was conducted in a Dutch Comprehensive Cancer Center certified by the Organisation of European Cancer Institutes (OECI accreditation) [36], the results of our study design also contribute to improving the external validity of the DIGEST in FEES.

Following the initial training program to measure the PAS and PPR in FEES, a first measurement attempt was made. When considering observer agreement of all bolus consistencies together, intraobserver agreement on the PAS was almost perfect and moderate to almost perfect for the PPR, whereas interobserver agreement on both the PAS and PPR was substantial. Interobserver agreement on the PPR per bolus consistency showed lower kappa values for thin liquid and bite-sized cracker (fair and moderate agreement). These lower kappa values were related to the PPR scores of observer 2, who presented a lower intraobserver agreement for all bolus consistencies than observer 1. After the additional training program, the overall intra and interobserver agreement (all bolus consistencies together) on the PPR improved during the second measurement attempt. Interobserver agreement on the safety, efficiency, and summary DIGEST grades was substantial to almost perfect. This is in line with previous research although the observers in these studies were experienced clinicians as opposed to our novice observers [18, 19].

Previous studies have described sufficient observer agreement on the PAS during FEES [6, 12, 37]. However, a comparison with the present study is not possible as observer agreement in these studies was not determined per bolus consistency and the populations were of mixed etiology also containing neurological patients.

As the pharyngeal residue rating scale used in the DIGEST is a newly described scale, there is no information in the literature on observer agreement on the PPR, with the exception of the DIGEST validation studies [18, 19]. While vallecular and pyriform sinus residue are usually scored separately, the PPR is scored based on the ‘overall’ pharyngeal residue measuring the percentage of the ingested bolus that remains in the entire pharynx after the first swallow. Furthermore, the PPR cannot be compared to the Yale Pharyngeal Residue Severity Rating Scale, which measures the percentage of site-specific pharyngeal space (vallecula or pyriform sinus) that is filled with bolus after the first swallow on that bolus [7]. Yet, measurement of overall pharyngeal residue may be more appropriate and reproducible compared to site-specific pharyngeal residue in this particular population of HNC patients. Alterations of the pharyngeal and/or laryngeal anatomy due to the tumor itself and/or the oncological treatment, including post-radiation edema and necessary surgical sacrifice of structures, can pose a challenge to precisely determine the anatomical location and estimate the amount of residue. Anatomical changes such as absence of an arytenoid or epiglottis following CO_2_ laser surgery for supraglottic larynx carcinoma, or post-radiation mucosal edema filling the vallecular and/or pyriform sinus space can make it very difficult to measure the amount of bolus residue at a specific anatomical subsite of the pharynx. Insufficient agreement on some DIGEST measurements, especially the PPR, during the first attempt of this experiment could also be explained by several other factors, such as the initial absence of clear definitions of cut-off values (boundaries) between ordinal categories of a scale and inexperience of the novice observer in determining the percentage of residue based on FEES images. For instance, during VFSS, the bolus volume is visible during all the swallowing phases. Therefore, the amount of bolus residue in the pharynx can be compared to the initial bolus volume in the oral cavity to facilitate the estimation of the proportion of bolus left in the pharynx after swallowing. As during FEES only the pharyngeal phase is shown, this comparison is not possible.

Improved observer agreement after the additional training program and the use of the manual support this reasoning. The additional training program and the manual with well-defined descriptions probably optimized the test conditions in terms of standardization of the measurements performed by the observers during the second measurement attempt, improving the reproducibility of the DIGEST measurements. This context-specific manual was based mainly on the difficulties experienced by the novice observers during the first measurement attempt in the present experiment. Therefore, the content of the manual cannot be extrapolated to different settings. Yet the use of the DIGEST under different conditions is encouraged, as this will contribute to its external validity.

Furthermore, bolus consistency can have an impact on the measurements in FEES exams [6, 10, 11]. For example, during the first measurement attempt, the interobserver agreement on the PPR was sufficient when analyzing all bolus consistencies together. However, interobserver agreement on the PPR was insufficient for thin liquid. The estimation of the amount of residue of thin liquid bolus can be challenging, since this less cohesive bolus spreads into the pharyngeal recesses more easily. Therefore, the percentage of thin liquid bolus remaining in the pharynx is more difficult to estimate compared to thick liquid, which is more cohesive when measured during the fork-drip test according to the IDDSI [34]. Bite-sized cracker also had an effect on observer agreement, as agreement for bite-sized cracker was often insufficient using linearly weighted kappa coefficient. This could be explained by the lack of variation of the scores across the PAS or PPR scales and the limited number of bolus swallows with bite-sized cracker. HNC patients frequently had incomplete dentition and/or severe xerostomia causing difficulty in mastication and swallowing of bite-sized cracker.

The findings of the present study were obtained using linearly weighted Cohen’s kappa coefficient to calculate observer agreement. Kappa is the most commonly reported measure of observer agreement in the medical literature [38]. During the second measurement attempt interobserver agreement on both PAS and PPR was not sufficient for bite-sized cracker (*κ* ≤ 0.55), yet the corresponding percentage of interobserver agreement on both PAS and PPR was high (≥ 82%). This statistical phenomenon, also called ‘first paradox’, of a high percentage of agreement between observers but low kappa values has been described extensively in the literature [39]. Kappa is a chance-corrected measure, but the level of agreement expected by chance alone is dependent on the distribution of marginal totals. Skewed distributions of scores across categorical scales can result in lower kappa values but this does not mean that the observer agreement is poor [40, 41].

Data collection and the DIGEST measurements of this study were performed prior to the publication of the study on the adaptation and validity of the DIGEST for FEES by Starmer et al. [19]. The design of the present study was based on the DIGEST protocol developed for VFSS, as published in the ‘original’ DIGEST study by Hutcheson et al. [18]. The measurements during the first measurement attempt were solely based on the information provided by the ‘original’ DIGEST study [18], and the insufficient observer agreement in our study showed the need for a more detailed description of the boundaries of each level of the ordinal variables. The ‘original’ DIGEST study determined interobserver agreement on the safety, efficiency, and summary DIGEST grades [18], yet our study also determined intra and interobserver agreement on the PAS and the PPR.

It is also important to emphasize that a videofluoroscopic measurement scale such as the DIGEST cannot be transformed directly, one-on-one into a FEES scale. Therefore, we also explored the criterion validity of the DIGEST in FEES by analyzing the correlation between the safety, efficiency, and summary DIGEST grade versus the EAT-10, FOIS, TNM, and MDADI. The EAT-10, FOIS, and MDADI were chosen as criterion measurements as they are patient-reported outcome measures (PROMs) which are part of the usual care protocol in our Comprehensive Cancer Center, representing different dimensions of swallowing impairment [42]. We found a significant correlation between the DIGEST efficiency grade versus the EAT-10 for both observers, implying that patients who presented increased levels of pharyngeal residue, had a higher level of self-perceived symptom severity on the EAT-10.

### Limitations of the Study

This study has some limitations. Only two observers were involved in our study. Results on observer agreement might have been different if a higher number of observers was included or if the degree of experience of the observers was different. We followed the original DIGEST protocol as described in the VFSS validation study [18] to the extent possible. However, different bolus consistencies and volumes were used in our study as data was collected in daily clinical practice using our standardized FEES protocol [9, 11, 31]. This may have led to different safety and efficiency grades and consequently to a different criterion validity. Next, the DIGEST only measures pharyngeal dysphagia. However, patients with isolated oral dysphagia with preservation of pharyngeal swallowing function, which is common in patients with carcinoma of the anterior mouth floor, will not be captured by the DIGEST. Furthermore, at the time of submission of the present study, a revised version of the DIGEST for VFSS (‘DIGEST version 2’) was published refining the measurement of the safety grade [43]. Yet both our study as well as prior research on the DIGEST [18, 19, 43] aim to improve the DIGEST, promoting wider use of the DIGEST by multiple professionals and also improve its external validity.

## Conclusion

The DIGEST showed to be a reproducible measurement for FEES in terms of observer agreement. However, agreement between novice observers on the DIGEST was only reached after specific observer-tailored training. Observer agreement should be analyzed by taking bolus consistency into account during training, as this might affect the interpretation of the outcome. A manual with well-defined descriptions can optimize the reproducibility of DIGEST measurements.

## Supplementary Information

Below is the link to the electronic supplementary material.

Supplementary file1 (DOCX 20 KB)

## Data Availability

The datasets generated during and/or analysed during the current study are available from the corresponding author on reasonable request.
