# Supplementary material for: Intra and interobserver agreement of the Dynamic Imaging Grade of Swallowing Toxicity Scale (DIGEST) in fiberoptic endoscopic evaluation of swallowing (FEES): the importance of observer-tailored training
Source: Eur Arch Otorhinolaryngol. 2023 Jan 27;280(6):2865–76. doi: 10.1007/s00405-023-07840-1 (PMC10175403; doi:10.1007/s00405-023-07840-1)
Supplement: Supplementary file 1 — Supplementary file1 (DOCX 20 KB) [file 405_2023_7840_MOESM1_ESM.docx]

**Supplementary Information**

**Online Resource 1** Flowchart of the training process of the novice observers
